# Maternal rubella immunity gaps in the vaccine era: a seroepidemiological study of pregnant women in Jiangsu, China

**DOI:** 10.3389/fimmu.2026.1971227

**Published:** 2026-09-24

**Authors:** Xiang Sun, Yuanyuan Zhu, Wen Wang

**Affiliations:** 1 Department of Expanded Program on Immunization, Jiangsu Provincial Center for Disease Control and Prevention, Nanjing, Jiangsu, China; 2 Department of Rheumatology and Immunology, Jiangsu Province (Suqian) Hospital, Suqian, Jiangsu, China

**Keywords:** birth cohort, maternal immunity, pregnant women, rubella, rubella IgG

## Abstract

**Background:**

Rubella elimination has substantially reduced virus circulation in China, but the changing pattern of immunity among women entering reproductive age remains unclear. We assessed the contemporary rubella immunity profile among pregnant women in Jiangsu Province.

**Methods:**

We conducted a cross-sectional serological study of 1,196 pregnant women in Jiangsu Province. Rubella-specific IgG antibodies were quantitatively measured. Seropositivity and antibody concentrations were compared across maternal age and birth cohorts. Restricted cubic spline (RCS) regression was used to assess nonlinear associations between age and immunity, and multivariable regression identified factors associated with seronegativity.

**Results:**

Overall rubella IgG seropositivity was 61.45%, with a geometric mean concentration of 24.21 IU/mL. Seropositivity increased from 37.77% among women aged ≤24 years to approximately 75% among women aged ≥30 years. Antibody concentrations showed a similar age-related gradient, with a nonlinear association between maternal age and rubella immunity. Younger birth cohorts consistently exhibited lower seropositivity and antibody concentrations, indicating a concentrated immunity gap among women entering reproductive age. Maternal age remained independently associated with rubella immunity, whereas gestational age was not.

**Conclusions:**

Despite sustained high RCV coverage and declining rubella transmission in China, substantial heterogeneity in maternal rubella immunity persists. Younger women and more recent birth cohorts may represent important susceptibility groups. Integrating maternal immunity surveillance with sustained childhood vaccination and preconception prevention strategies may help maintain rubella elimination and prevent congenital rubella syndrome.

## 1 Background

Rubella is a vaccine-preventable viral infection of particular public health importance because infection during pregnancy, especially in early gestation, can cause congenital rubella syndrome (CRS), including congenital heart defects, hearing impairment, and other permanent disabilities. Maintaining adequate immunity among women of reproductive age is therefore essential for sustaining rubella elimination and preventing CRS (1–4).

China has made substantial progress in rubella control through the expansion of rubella-containing vaccine (RCV) use. RCV was incorporated into the national immunization program in 2008, and coverage of the first and second routine doses has remained above 95% since 2012. This sustained vaccination program has been accompanied by a substantial decline in rubella incidence nationwide (4, 5). However, the epidemiological transition toward rubella elimination may create new challenges for population immunity. As circulation of wild-type rubella virus decreases, younger generations increasingly rely on vaccine-induced rather than infection-derived immunity, while opportunities for natural immune boosting are reduced (6, 7). Consequently, susceptible individuals may accumulate in successive birth cohorts, potentially creating immunity gaps among women entering reproductive age (8).

Pregnant women provide an important population in which to evaluate these changing patterns of rubella immunity because insufficient maternal immunity represents a direct vulnerability for CRS prevention. Previous studies in China have largely focused on rubella surveillance and vaccination coverage, whereas contemporary quantitative assessments of rubella immunity among pregnant women remain limited (8–10). In particular, it is unclear whether immunity differs substantially according to maternal age and birth cohort, and whether younger women entering reproductive age may constitute a population with insufficient antibody protection. Characterizing both rubella IgG seropositivity and quantitative antibody concentrations may therefore provide a more informative assessment of the maternal immunity landscape than serostatus alone (6).

Jiangsu Province provides an appropriate setting in which to investigate these questions. As one of China’s economically developed provinces with established immunization and infectious disease surveillance systems, Jiangsu has experienced the broader epidemiological transition associated with China’s successful rubella vaccination program (4). Nevertheless, the current serological immunity profile of pregnant women in the province and the distribution of potential immunity gaps across maternal age and birth cohorts remain unclear.

Therefore, this study aimed to characterize the contemporary rubella immunity landscape among pregnant women in Jiangsu Province using quantitative rubella IgG serology. We specifically assessed overall seroprevalence and antibody concentrations, investigated nonlinear associations between maternal age and rubella immunity, examined differences across birth cohorts, and identified factors associated with seronegativity. We further characterized the distribution of immunity gaps across age and birth cohorts and interpreted these findings in the context of the changing rubella epidemiology in Jiangsu and China’s broader transition toward rubella elimination. By linking maternal serological immunity with the historical evolution of rubella vaccination and disease transmission in China, this study sought to identify populations that may remain vulnerable during pregnancy and provide evidence to inform strategies for sustaining rubella elimination and preventing CRS.

## 2 Materials and methods

### 2.1 Study design and population

This cross-sectional seroepidemiological study was conducted among pregnant women receiving routine antenatal care in Jiangsu Province, eastern China. Pregnant women in the third trimester were prospectively recruited in 2024. Eligible participants met the following inclusion criteria (1): gestational age between 30 and 41 weeks at blood sampling (2); availability of complete demographic and clinical information, including maternal age and gestational weeks; (3) availability of serum samples for quantitative measurement of rubella IgG antibodies.

Women with severe pregnancy complications, active infectious diseases, immunodeficiency disorders, or a history of blood product transfusion during pregnancy were excluded. A total of 1,196 pregnant women were included in the final analysis. The study protocol was reviewed and approved by the institutional ethics committee of Jiangsu Provincial Center for Disease Control and Prevention (approval No. JSJK2023-B027-02). Written informed consent was obtained from all participants before enrollment. In addition, reported rubella surveillance data in Jiangsu Province during 2015–2025 were retrieved from the China National Notifiable Disease Reporting System (NNDRS) to describe temporal epidemiological trends and provide contextual interpretation of maternal immunity gaps in the vaccine era.

### 2.2 Serological assessment of rubella immunity

Peripheral venous blood samples were collected during routine antenatal visits in the third trimester.

Serum samples were separated and stored according to standard laboratory procedures before antibody testing.

Rubella virus-specific IgG antibodies were quantified using a commercial enzyme-linked immunosorbent assay (ELISA) according to the manufacturer’s instructions (Virion\Serion, Germany). Antibody concentrations were expressed as international units per milliliter (IU/mL). Based on established clinical criteria, rubella IgG concentration ≥10 IU/mL was defined as seropositive, indicating evidence of protective immunity, whereas concentrations below this threshold were considered seronegative and potentially susceptible to rubella infection. Because antibody concentrations demonstrated a skewed distribution, continuous IgG levels were log-transformed before regression analyses and summarized as geometric mean concentrations (GMCs) with corresponding 95% confidence intervals.

### 2.3 Definition of demographic variables and birth cohorts

Maternal age was analyzed both as a continuous variable and categorical variable. For descriptive analysis, participants were categorized into four age groups:

≤24 years, 25–29 years, 30–34 years and ≥35 years. To evaluate historical changes in rubella immunity patterns, birth cohort was calculated based on maternal age at enrollment and categorized into sequential birth cohorts representing different epidemiological and vaccination eras:≤1985,1986–1990,1991–1995,1996–2000 and ≥2001.These birth cohorts were used to explore potential differences in immunity profiles associated with changing patterns of natural infection exposure and vaccine-derived immunity.

### 2.4 Assessment of maternal immunity gap

The population immunity gap was defined as the proportion of pregnant women lacking protective rubella antibodies:

Immune gap(%)=1−Rubella IgG seropositivity rate

Immune gaps were evaluated according to: maternal age groups; birth cohorts; age–birth cohort combinations. A heatmap visualization was generated to identify population subgroups with increased susceptibility to rubella infection.

### 2.5 Statistical analysis

Continuous variables were summarized as mean ± standard deviation (SD) or median with interquartile range (IQR), as appropriate, while categorical variables were presented as numbers and percentages. Differences in rubella IgG seropositivity across maternal age groups and other categorical variables were assessed using the chi-square test or Fisher’s exact test, when appropriate. Trends across ordered groups were evaluated using the Cochran–Armitage trend test. Rubella IgG concentrations were log-transformed for regression analyses and summarized as GMCs with 95% confidence intervals (CIs). Multivariable logistic regression models were performed to identify factors associated with rubella seronegativity, with adjusted odds ratios (aORs) and 95% CIs estimated. RCS regression models were applied to assess potential nonlinear associations between maternal age and rubella antibody levels, adjusting for gestational age. Birth cohort-specific differences in rubella immunity were evaluated by comparing seropositivity and antibody concentrations across predefined birth cohorts. The population immunity gap was calculated as the proportion of women with rubella IgG concentrations below the protective threshold and was visualized using heatmaps. All statistical analyses were performed using R software. All statistical tests were two-sided, and P values <0.05 were considered statistically significant.

## 3 Result

### 3.1 Baseline characteristics and rubella immunological profiles

A total of 1,196 pregnant women were enrolled in this study. The mean maternal age was 28.7 ± 4.8 years (median, 28 years). Overall, 19.5% of participants were aged ≤24 years, 38.3% were 25–29 years, 31.4% were 30–34 years, and 10.8% were aged ≥35 years. Blood samples were collected at a median gestational age of 34 weeks. The overall rubella IgG seropositivity rate was 61.45% (95% CI: 58.68%–64.22%), and the geometric mean concentration (GMC) across the entire cohort was 24.21 IU/mL (95% CI: 22.88–25.55).

When stratified by maternal age, both seropositivity rates and antibody titers demonstrated a significant upward trend (*P*_trend_ < 0.001). Pregnant women aged ≤24 years exhibited the lowest seropositivity rate at 37.77% (95% CI: 31.54%–43.99%) and a mean titer of 14.45 IU/mL (95% CI: 12.02–16.89). This rate rose to 57.86% (95% CI: 53.33%–62.39%) in the 25–29 group, 75.80% (95% CI: 71.47%–80.13%) in the 30–34 group, and remained high at 75.19% (95% CI: 67.72%–82.66%; mean titer: 29.25 IU/mL, 95% CI: 25.38–33.11) among those aged ≥35 years. In contrast, rubella immunity profiles remained stable across the gestational spectrum. Seropositivity rates fluctuated non-linearly between 59.02% and 67.45% across different gestational intervals, with no statistically significant differences or trends observed (p=0.245) (**Table 1**).

**Table 1:** Baseline characteristics and rubella antibody seroprevalence among 1,196 pregnant women.

Characteristics	Total enrolled (N=1,196)	Seropositive n (%)	95% CI of seropositivity	GMC (IU/mL) (95% CI)*	P-value/Ptrend​
Overall	1,196	735 (61.45%)	58.66% – 64.17%	24.21 (22.88 – 25.55)	–
Maternal Age (years)					<0.001
≤24	233	88 (37.77%)	31.79% – 44.14%	14.45 (12.03 – 16.88)	
25–29	458	265 (57.86%)	53.29% – 62.30%	21.96 (19.91 – 24.02)	
30–34	376	285 (75.80%)	71.22% – 79.85%	31.28 (28.74 – 33.82)	
≥35	129	97 (75.19%)	67.08% – 81.85%	29.25 (25.42 – 33.07)	
Gestational Weeks					0.245
30.0–31.9	326	195 (59.82%)	54.41% – 64.99%	22.41 (20.05 – 24.77)	
32.0–33.9	254	171 (67.32%)	61.33% – 72.80%	26.97 (23.92 – 30.01)	
34.0–35.9	89	57 (64.04%)	53.69% – 73.24%	24.41 (19.87 – 28.95)	
36.0–37.9	466	276 (59.23%)	54.71% – 63.60%	23.91 (21.73 – 26.09)	
≥38.0	61	36 (59.02%)	46.50% – 70.46%	24.41 (18.20 – 30.61)	

### 3.2 Age and birth cohort effects on maternal rubella immunity

To elucidate the complex epidemiological dynamics shaping maternal rubella immunity, we evaluated the non-linear relationship between maternal age and antibody concentrations, alongside stratified birth cohort profiles (**Figure 1**).

**Figure 1:**
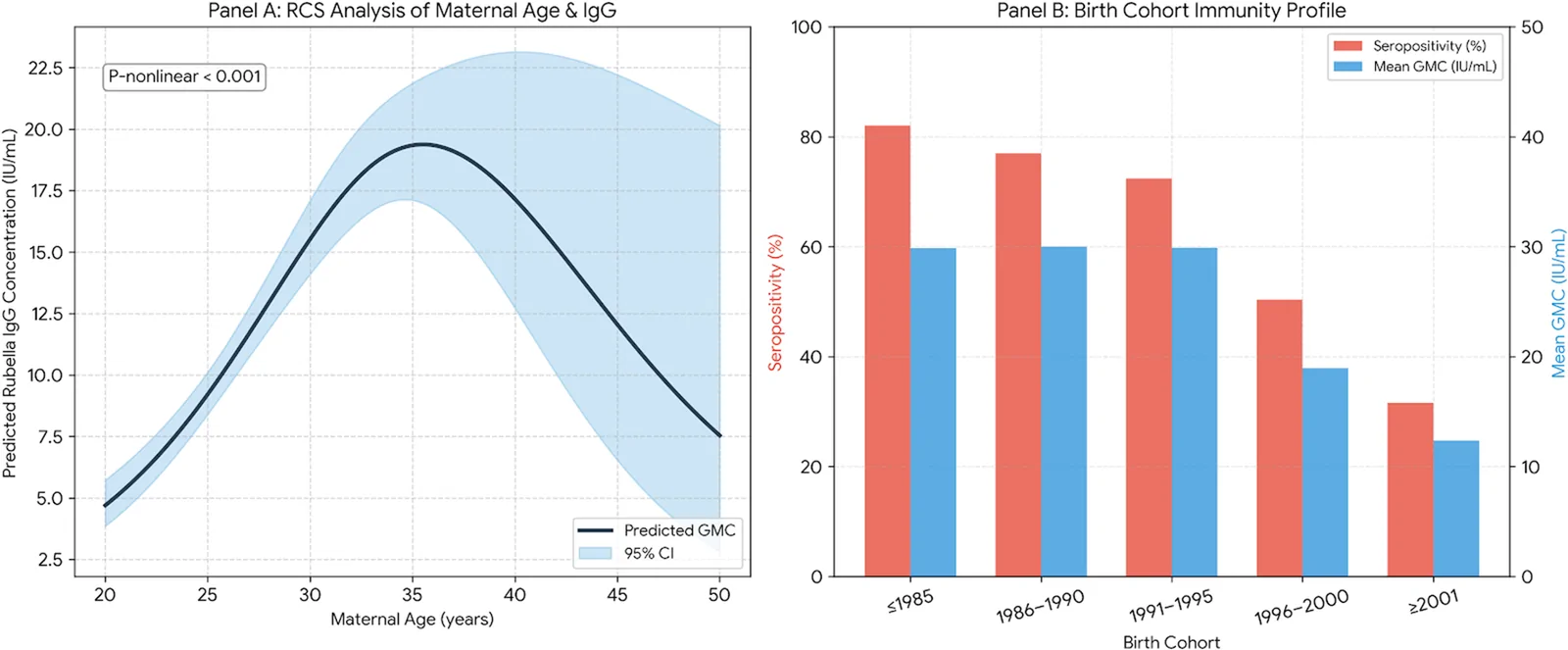
Age and birth cohort effects on maternal rubella immunity. **(A)** RCS Analysis of Maternal Age & IgG; **(B)** Birth Cohort Immunity Profile.

As demonstrated in Panel A, restricted cubic spline (RCS) regression—adjusted for gestational age—revealed a highly significant non-linear association between maternal age and predicted rubella IgG concentrations (*P*_nonlinear_ < 0.001). The predicted antibody titer exhibited an upward trajectory from early adulthood, peaking among gravidas aged approximately 35 years, before exhibiting a downward trend in older age brackets.

To further dissect this phenomenon across generational gradients, participants were categorized into five distinct birth cohorts (Panel B). A stark generational decline in both seropositivity and geometric mean concentrations (GMC) was observed among younger cohorts. Women born in ≤1985 and 1986–1990 maintained robust seropositivity rates of 82.1% and 77.0%, respectively. In contrast, seropositivity plummeted precipitously in younger generations, dropping to 50.4% in the 1996–2000 birth cohort and reaching a nadir of just 31.6% among the most recent childbearing-aged women (≥2001 cohort; mean GMC dropped correspondingly to 12.4 IU/mL).

### 3.3 Determinants of insufficient rubella immunity among pregnant women

Multivariable regression models were applied to identify independent predictors of maternal rubella immunity. Maternal age remained the strongest determinant of both seropositivity status and antibody concentration after adjustment for gestational age.

In logistic regression analysis, each one-year increase in maternal age was associated with a 13.7% higher likelihood of rubella seropositivity (adjusted OR [aOR], 1.137; 95% CI, 1.107–1.168; P<0.001). Similarly, maternal age was independently associated with higher quantitative antibody levels in linear regression models (β=0.074; 95% CI, 0.059–0.089; P<0.001).

In contrast, gestational age was not independently associated with either rubella seropositivity (aOR, 0.976; 95% CI, 0.934–1.020) or antibody concentration, suggesting that antibody variability observed among participants primarily reflected differences in underlying immune history rather than short-term changes during late pregnancy (**Table 2**).

**Table 2:** Multivariable regression analyses of factors associated with maternal rubella seropositivity and antibody concentration.

Variables	Multivariable logistic regression (for seropositivity)		Multivariable linear regression (for log-GMC)	
	Adjusted OR (95% CI)	P-value	β Coefficient (95% CI)	P-value
Intercept	0.097 (0.018 – 0.523)	0.007	0.644 (-0.383 – 1.671)	**0.219**
Maternal Age (per year increase)	1.137 (1.107 – 1.168)	< 0.001	0.074 (0.059 – 0.089)	**< 0.001**
Gestational Weeks (per week increase)	0.976 (0.934 – 1.020)	0.285	-0.008 (-0.035 – 0.019)	**0.579**

OR, Odds Ratio; CI, Confidence Interval; β, Standardized regression coefficient. Bold values indicate statistical significance (P<0.05).

### 3.4 Maternal immunity gaps in the context of rubella elimination

The integration of population-level surveillance trends and individual-level sero-epidemiological profiles reveals a critical public health phenomenon in the endgame of rubella elimination in Jiangsu Province (**Figures 2**, **3**).

**Figure 2:**
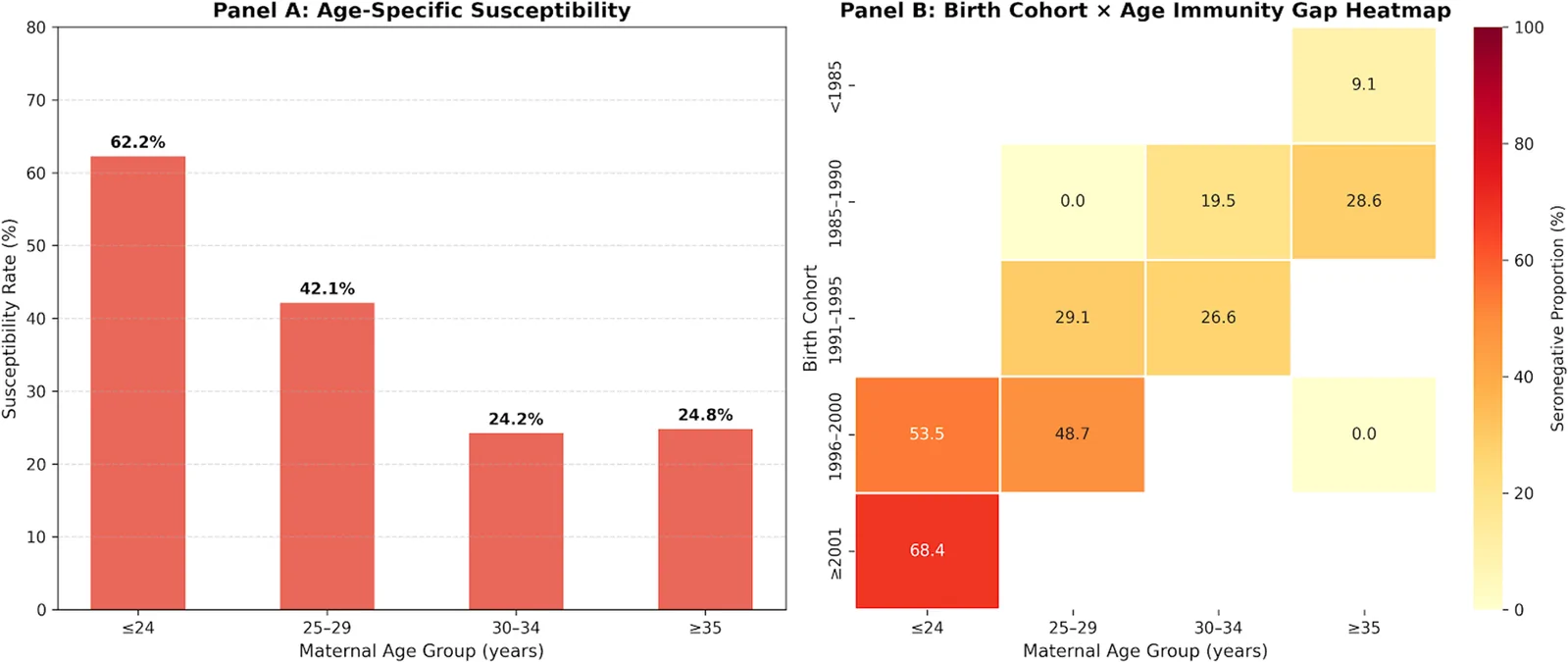
Maternal rubella immunity gap landscape. **(A)** Age‐Specific Susceptibility; **(B)** Birth Cohort × Age Immunity Gap Heatmap.

**Figure 3:**
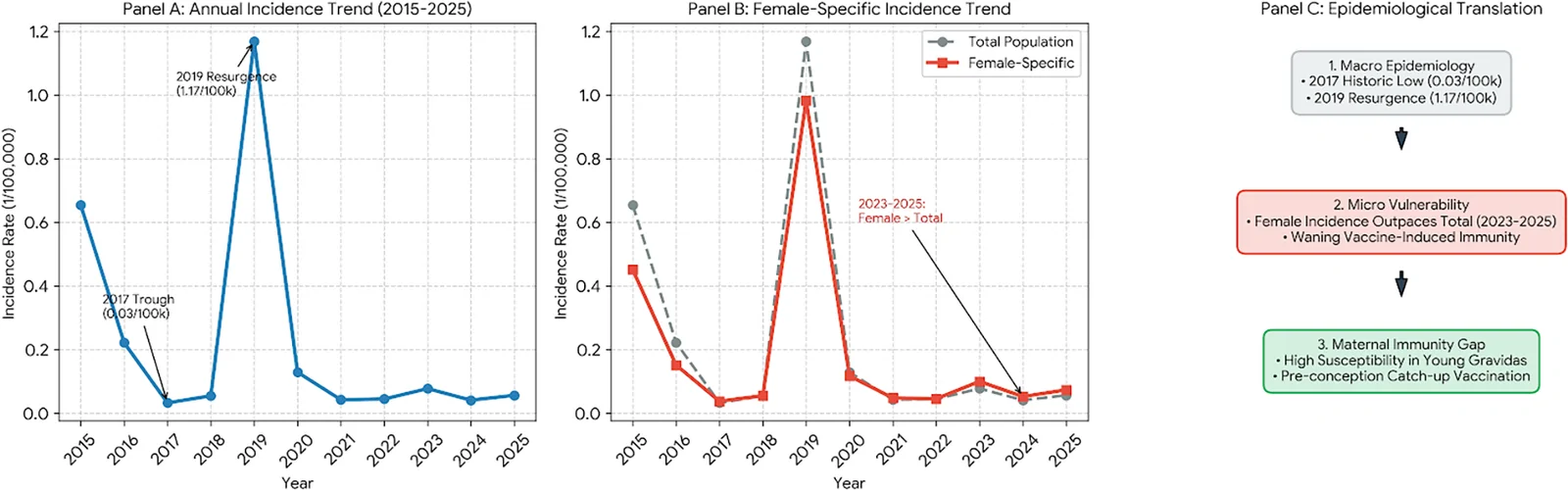
Rubella epidemiological transition and maternal immunity vulnerability in Jiangsu Province. **(A)** Annual Incidence Trend (2015‐2025); **(B)** Female‐Specific Incidence Trend; **(C)** Epidemiological Transition .

As demonstrated by the 2015–2025 surveillance data (**Figures 3A, B**), rubella incidence underwent a drastic epidemiological transition characterized by a historic low in 2017 (0.03 per 100,000) followed by a prominent regional resurgence in 2019 (1.17 per 100,000), subsequently entering a sustained post-epidemic low-transmission era. While this macro-level suppression successfully interrupted widespread pediatric circulation, our conceptual framework and micro-level data indicate that it inadvertently precipitated a generational vulnerability shift (**Figure 3C**).

Specifically, the cessation of wild-type viral exposure has deprived modern childbearing-aged cohorts of natural immunological boosting. Consequently, an escalating susceptibility gradient is concentrated among younger generations (**Figure 2**). Age-specific susceptibility peaked at 62.2% among gravidas aged ≤24 years (**Figure 2A**). Furthermore, the birth cohort × age interaction heatmap (**Figure 2B**) explicitly identifies that the largest immunity gaps reside in the youngest strata—reaching 68.4% in the ≥2001 birth cohort (≤24 years) and 48.7–53.5% in the 1996–2000 birth cohort.

These findings underscore that while universal childhood immunization has effectively controlled acute pediatric infections, the resulting absence of natural booster stimuli has generated a profound population immunity gap among young gravidas, shifting the primary rubella-associated risk from childhood outbreaks to maternal-neonatal vulnerability.

## 4 Discussion

In this study of 1,196 pregnant women in Jiangsu Province, we identified a substantial gap and marked heterogeneity in maternal rubella immunity despite the sustained high coverage of RCV in China. Overall rubella IgG seropositivity was 61.45%, with a GMC of 24.21 IU/mL. Maternal immunity varied substantially by age, with younger women showing considerably lower seropositivity and antibody concentrations than older women. Restricted cubic spline analysis further demonstrated a nonlinear association between maternal age and antibody levels, while birth cohort analysis revealed a clear generational pattern, with lower immunity observed among more recent cohorts. Maternal age remained independently associated with both rubella seropositivity and antibody concentration, whereas gestational age was not. Taken together, these findings indicate that the current maternal rubella immunity profile is not homogeneous and that younger women entering reproductive age may represent an important population with insufficient protection (11–13).

The observed immunity gap should be interpreted in the context of China’s substantial progress in rubella control. Since RCV was incorporated into the national immunization program in 2008, sustained high vaccination coverage has contributed to a dramatic reduction in rubella transmission (4, 5, 14). This achievement has undoubtedly reduced the risk of rubella infection and represents an important step toward elimination. However, successful reduction in virus circulation also changes the way population immunity is acquired and maintained (6, 15, 16). Before widespread vaccination, natural infection contributed substantially to rubella immunity, whereas younger generations have increasingly relied on vaccination as their primary source of protection. With declining circulation of wild-type virus, opportunities for natural exposure and immune boosting are likely to have become less frequent (17). Consequently, the immunity profile of successive generations may increasingly depend on the persistence of vaccine-derived protection. Our finding of lower immunity among younger birth cohorts is consistent with this broader epidemiological transition, although the cross-sectional nature of our study does not allow us to determine the relative contributions of vaccination history, previous infection, antibody persistence, or reduced natural boosting (18, 19).

The age-related pattern observed in this study is particularly noteworthy. Seropositivity increased from 37.77% among women aged ≤24 years to approximately 75% among women aged 30 years or older, and quantitative antibody concentrations showed a similar gradient. The nonlinear pattern identified by RCS analysis further suggests that maternal immunity cannot be adequately characterized by a simple linear relationship with age. Older women may have accumulated immunity through both vaccination and natural infection during periods when rubella circulation was greater, whereas younger women were more likely to grow up in an era of high vaccination coverage and substantially reduced virus circulation (6, 15, 20). Thus, the higher antibody levels observed among older women should not necessarily be interpreted as evidence that immunity naturally increases with age; rather, they may reflect differences in historical exposure and vaccination environments across generations. Importantly, we did not observe a significant association between gestational age and either seropositivity or antibody concentration, suggesting that the major differences in immunity were more likely related to underlying immune history than to short-term changes during late pregnancy (18, 21).

The birth cohort findings provide further evidence that maternal immunity is changing across generations. The progressively lower seropositivity and antibody concentrations observed among younger cohorts suggest that the immunity gap is concentrated rather than uniformly distributed across women of reproductive age (22–24). This has important implications for rubella elimination because reported incidence alone may underestimate the underlying susceptibility of the population when transmission is very low. Although the reduction in rubella circulation is a major public health success, the persistence of susceptible women may remain relevant if sporadic transmission or virus importation occurs. For rubella, even limited transmission can have disproportionate consequences when infection occurs during pregnancy because the principal public health outcome of concern is CRS rather than severe maternal disease (25). Therefore, maintaining immunity among women before pregnancy remains an important component of the elimination strategy.

These findings suggest that maternal serological surveillance may provide valuable complementary information to routine vaccination coverage and case-based surveillance. The identification of lower immunity among younger women does not necessarily indicate failure of the childhood vaccination program. Rather, it highlights the distinction between maintaining high vaccination coverage in children and ensuring sufficient immunity when successive cohorts reach reproductive age. Preconception health services may provide an opportunity to identify women without evidence of rubella immunity and to consider appropriate vaccination before pregnancy according to national recommendations (8, 21). At the population level, continued monitoring of immunity among women of reproductive age, particularly younger birth cohorts, may help detect emerging susceptibility before it translates into increased maternal infections or CRS. Such strategies should complement rather than replace the existing high-coverage childhood immunization program.

This study has several limitations. First, the cross-sectional design precludes causal inference regarding the reasons for lower immunity among younger women. Individual vaccination histories, vaccination dates and doses, previous rubella infection, and longitudinal antibody measurements were unavailable, preventing direct assessment of vaccine-derived antibody persistence or the contribution of natural boosting. Second, participants were pregnant women recruited during the third trimester and may not fully represent all women of reproductive age in Jiangsu. Third, rubella IgG concentration reflects humoral immunity but is not an absolute surrogate for protection against infection, and cellular immunity was not assessed. Finally, birth cohort differences should be interpreted as epidemiological patterns rather than causal cohort effects.

In conclusion, our findings demonstrate substantial heterogeneity in rubella immunity among pregnant women in Jiangsu Province, with the greatest immunity gaps concentrated among younger women and more recent birth cohorts. In the context of China’s successful reduction in rubella transmission, these findings highlight a potentially important shift in the epidemiology of susceptibility from childhood toward women entering reproductive age. Sustaining rubella elimination will therefore require not only continued high childhood RCV coverage but also surveillance of maternal immunity and attention to susceptible women before pregnancy. Closing these immunity gaps may be important for maintaining the gains achieved through China’s rubella vaccination program and for preventing CRS as the country progresses toward rubella elimination.

## Data Availability

The raw data supporting the conclusions of this article will be made available by the authors, without undue reservation.
